# Comparison of clinical manifestations and antibiotic resistances among three genospecies of the *Acinetobacter calcoaceticus-Acinetobacter baumannii* complex

**DOI:** 10.1371/journal.pone.0191748

**Published:** 2018-02-01

**Authors:** Lu Chen, Juxiang Yuan, Yingjun Xu, Fengxia Zhang, Zhenlei Chen

**Affiliations:** 1 College of Public Health, North China University of Science and Technology, Tangshan, Hebei, China; 2 Department of Infection Control, The Second Hospital of Tangshan, Tangshan, Hebei, China; 3 Department of Infection Control, Kailuan General Hospital, Tangshan, Hebei, China; Northwestern University Feinberg School of Medicine, UNITED STATES

## Abstract

The *Acinetobacter calcoaceticus*-*Acinetobacter baumannii* (ACB) complex has emerged as a high priority among hospital-acquired pathogens in intensive care units (ICUs), posing a challenge to infection management practices. In this study, the clinical characteristics, antimicrobial susceptibility patterns, and patients outcome among genospecies were retrospectively compared. Samples were taken from the tracheal secretions of 143 patients in the ICU. Genospecies of the ACB complex were discriminated by analysis of the 16S-23S rRNA gene intergenic spacer (ITS) sequence. Univariate and multiple variable logistic regression analyses were performed to identify risk factors for infection and mortality. Three genospecies were isolated: *A*. *baumannii* (73, 51.0%), *A*. *nosocomialis* (29, 20.3%), and *A*. *pittii* (41, 28.7%). The results showed that the distribution of infection and colonization among the three genospecies were the same, while *A*. *baumannii* was more resistant to common antibiotics than *A*. *nosocomialis* and *A*. *pittii*. Advanced age, a long stay in the ICU, acute physiology and chronic health evaluation (APACHE) II score, the use of a mechanical ventilator, and previous antibiotic use were risk factors for patient infection. The APACHE II score was a risk factor for mortality in patients with ACB complex isolated from tracheal secretions. Poor outcome of patients with ACB complex isolated from tracheal secretion appears to be related to the APACHE II score rather than genospecies.

## Introduction

*Acinetobacter baumannii*, *Acinetobacter pittii* (formerly *Acinetobacter* genospecies 3), and *Acinetobacter nosocomialis* (formerly *Acinetobacter* genospecies 13TU) are common clinical genospecies of the *Acinetobacter calcoaceticus-Acinetobacter baumannii* (ACB) complex. They are gram-negative and genetically highly related, making it difficult to distinguish them phenotypically with routine laboratory methods [1]. In intensive care units (ICUs), most ACB complex bacteria are isolated from tracheal secretions and blood samples. Unlike ACB complex bacteria detected in the blood, which all cause infections, clinical manifestations caused by ACB complex bacteria isolated from tracheal secretions can be divided into those that cause infection and those that colonize. Although the clinical impacts of different ACB complex genospecies on the severity of bacteraemia have been reported [2–5], little information is available on the clinical manifestation of different ACB complex genospecies isolated from tracheal secretion samples. Many ACB complex bacteria isolated from the ICU are antibiotic resistant, including multidrug resistant and extensively drug resistant isolates [6, 7]. Studies on the correlations between ACB complex genospecies isolated from blood and antibiotic resistance have been conducted [2–5]. Little information is available on the correlations between ACB complex genospecies isolated from tracheal secretions and antibiotic resistance. In this study, 16S-23S rRNA gene ITS sequence analysis was used for identifying ACB complex genospecies isolated from ICU patients’ tracheal secretions, and the clinical characteristics, antimicrobial susceptibility patterns, and patient outcomes among genospecies were compared.

## Materials and methods

### Setting, study design and patient eligibility

We conducted a retrospective review of electronic medical records at three tertiary hospitals, which were affiliated Hospital of North China University of Science and Technology, the Tangshan Gongren Hospital, and the Second Hospital of Tangshan, located in northern China with 980, 1100, and 1300 beds, respectively, and a 20–30-bed general ICU in each hospital. From January to December 2013, ACB complex bacteria isolated from ICU patients’ tracheal secretion samples were collected and genotyped. The patients’ clinical manifestations, the antimicrobial susceptibility patterns of the isolates, and the risk factors for infection and mortality were assessed retrospectively and compared among the different genospecies of the ACB complex. Patient eligibility inclusion criteria included (a) admission to the ICU for medical or surgical treatments, (b) a stay in the ICU for more than 48 hours before ACB complex isolation, and (c) ACB complex isolated from tracheal secretion samples. Exclusion criteria included (a) age < 18 years old, (b) patients with co-pathogen or co- genospecies isolation, or (c) patients who refused to participate in the study.

### Laboratory testing

In each of the three hospitals, respiratory secretions were collected from patients receiving preventive tracheotomy or ventilator support; sputum were collected in the morning, after the patient gargled repeatedly with fresh water, the patient coughed up deep sputum from the respiratory tract, which was then collected. The Siemens MicroScan® WalkAway 96 PLUS instrument (Dade Behring Inc., West Sacramento, CA, USA) was used for the identification of ACB complex bacteria and drug sensitivity analyses. All susceptibilities were interpreted according to Clinical and Laboratory Standards Institute guidelines [8]. Susceptibility to tigecycline was determined by US FDA breakpoints for Enterobacteriaceae [9]. ACB complex with tigecycline MIC values ≤2 mg/L were considered susceptible.

Amplification of the 16S-23S rRNA gene ITS region and nucleotide sequence determination were used for ACB complex genospecies identification [10]. Primers 1512F (5′GTCGTAACAAGGTAGCCGTA3′) and 6R (5′GGGTTYCCCCRTTCRGAAAT3′) (where Y is C or T and R is A or G) were used to amplify a DNA fragment. Single amplicons were subjected to direct nucleotide sequencing (Sangon Biotech, Shanghai, China). For strains with multiple ITS fragments, the ITSs were cloned and then sequenced after reamplification. The portions of the 16S and 23S rRNA gene regions were removed from the sequence data to obtain the exact ITS sequences, and then the sequences were analysed using the Basic Local Alignment Search Tool (BLAST). The identified genospecies were classified as *A*. *baumannii*, *A*. *nosocomialis*, or *A*. *pittii*.

### Clinical characteristics and outcome analysis

We reviewed the electronic medical records of eligible patients to collect clinical information, including demographic data, reasons for admission to the ICU, infection or colonization, admission APACHE II score, previous antibiotic use, length of stay in the ICU before ACB complex detection, use of a mechanical ventilator, and 30-day mortality.

### Definitions

Multidrug resistance is defined as non-susceptibility to at least one agent in three or more antimicrobial categories, including aminoglycosides, antipseudomonal carbapenems, antipseudomonal fluoroquinolones, antipseudomonal penicillins+β-lactamase inhibitors, extended-spectrum cephalosporins, folate pathway inhibitors, penicillins+β-lactamase inhibitors, polymyxins and tetracyclines [11]. The clinical criteria for infection were established by the Centers for Disease Control and Prevention [12]. Patients with detectable ACB complex bacteria from tracheal secretions meeting at least one of the following criteria were diagnosed as having bacterial infections: (1) patient had clinical evidence of infection such as fever of 38°C, leukopenia <4000 WBC/mm^3^ or leukocytosis ≥12000 WBC/mm^3^, cough, new or increased sputum production, rhonchi, wheezing, or worsening gas exchange or (2) patient had radiographic evidence of infection such as infiltrate, consolidation or an abscess cavity. Patients with detectable ACB complex bacteria from tracheal secretions with no clinical and radiographic evidence of respiratory infection were diagnosed as having bacterial colonization. Previous antibiotic use was defined as treatment for at least 24 hours within the 30 days prior to ACB complex isolation. Mortality was defined as a death occurring within 30 days after isolation of ACB complex bacteria.

### Statistics

Statistical analyses were performed with SPSS 16.0 (SPSS Inc., Chicago, IL, USA). Continuous variables were compared with Student’s *t* test or one-way analysis of variance for normally distributed variables and the Mann-Whitney *U*-test for non-normally distributed variables. Categorical variables were expressed as the percentage of the total number of patients and were compared with the χ^2^ test or Fisher’s exact test, as appropriate. Pairwise comparisons were performed, and a modified Bonferroni-adjusted α was used for pairwise comparisons if the overall comparison among the three genospecies was statistically significant. Variables with *p* values < 0.10 in the univariate analysis were included in the multiple variable logistic regression analysis with backward stepwise procedures based on the maximum partial likelihood estimates to construct a model. The Hoshmer-Lemeshow goodness-of-fit test was used to assess whether a multiple variable logistic regression model was fit. A model with *p*-value > 0.05 of Hoshmer-Lemeshow’s test was considered as fit for the multiple logistic regression. All tests were two-tailed, and a *p* value < 0.05 was considered significant.

### Ethics approval

The study protocol was approved by the ethics committees of the three hospitals that participated in this study (reference number: TSEY-YL-201209). All aspects of the study complied with the ethical standards of the 1964 Declaration of Helsinki and its later amendments. Written consent was obtained from all individual participants. In the course of the research, there was no access to any information that could potentially identify individual patients.

## Results

**Fig 1:**
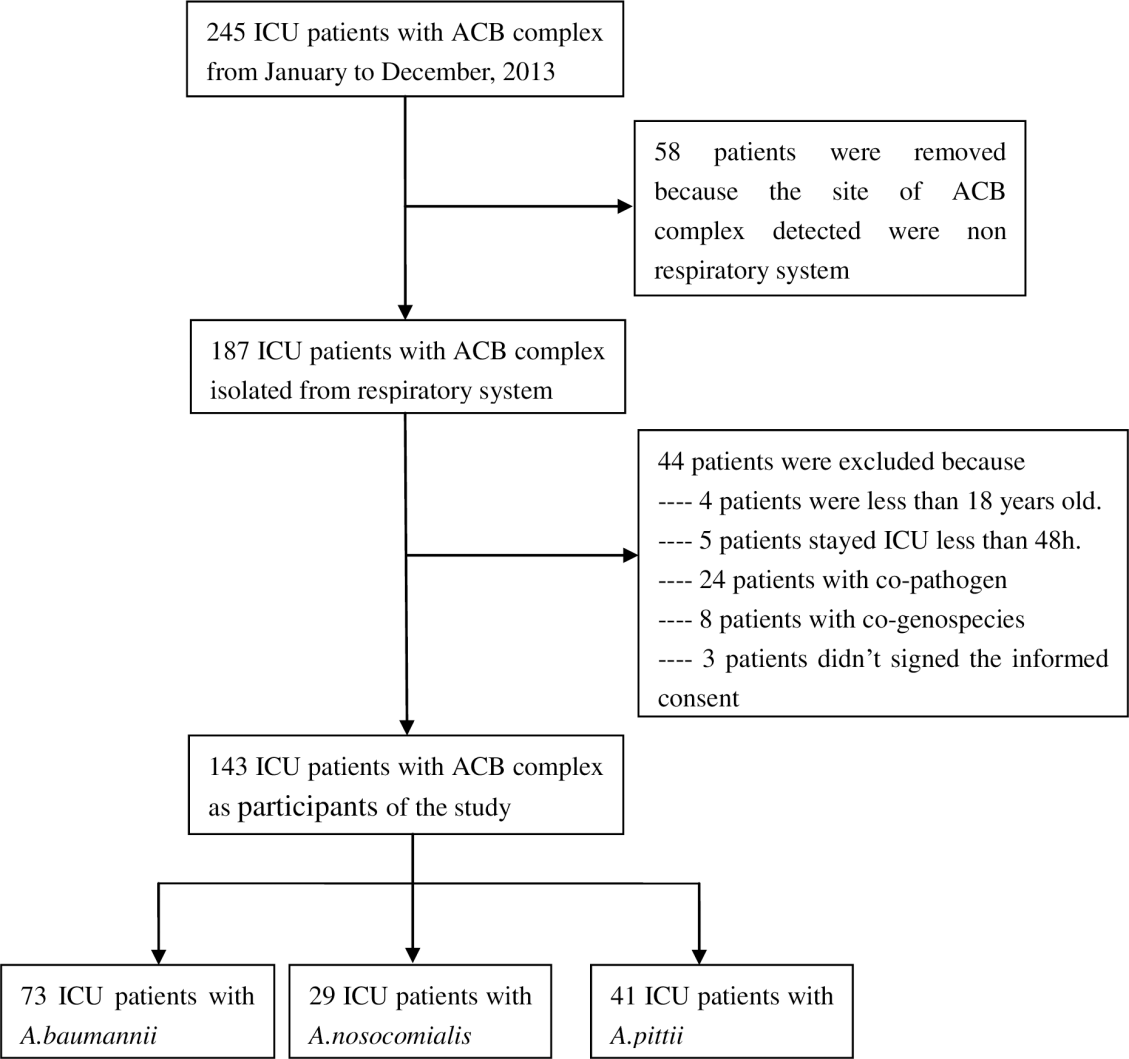
Flowchart of the study enrollment.

One hundred forty-three ICU patients with ACB complex isolated from tracheal secretion samples became participants in the study, excluding those who did not fulfil the inclusion criteria (Fig 1). 16S-23S rRNA gene ITS sequence analysis was used for ACB complex genospecies identification [10]. As a result, three ACB complex genospecies were isolated, including *A*. *baumannii* (73, 51.0%), *A*. *nosocomialis* (29, 20.3%), and *A*. *pittii* (41, 28.7%).

Results from analyses of demographic characteristics, seasonality, clinical manifestations, infection risk factors, and patient outcomes with ACB complex data are shown in Table 1. There were no significant differences in age (*p* = 0.300), gender (*p* = 0.791), seasonal distribution (*p* = 0.993), admitting diagnosis to the ICU (*p* = 0.406), APACHE II score (*p* = 0.754), previous antibiotic use (*p* = 0.114), length of stay in the ICU before ACB complex isolation (*p* = 0.671), or use of mechanical ventilator (*p* = 0.070). Among patients with the three genospecies, *A*. *baumannii*, *A*. *nosocomialis* and *A*. *pittii* exhibited a similar distribution of infection and colonization (*p* = 0.066). There was no significant difference in mortality among the patients carrying the three genospecies (*p* = 0.445).

**Table 1:** Comparisons of demographic characteristics and clinical manifestations among patients with different genospecies of ACB complex isolated from tracheal secretions.

Demographic characteristics and clinical manifestations	A. baumannii, n = 73, n (%)	A.nosocomialis, n = 29, n (%)	A. pittii, n = 41, n (%)	Statistical analysis (*P* value)			
				overall	A.baumannii versus A. nosocomialis	A. nosocomialis versus A. pittii,	A.baumannii versus A. pittii,
**Age, years(mean** **± SD)**	60.79 ±18.651	65.24 ±14.682	58.44 ±19.117	0.300	-	-	-
**Gender**							
**Male**	43(58.9)	19(65.5)	26(63.4)				
**Female**	30(41.1)	10(34.5)	15(36.6)	0.791	-	-	-
**Seasonal distribution**							
**Spring**	14(19.1)	5(17.2)	9(22.0)				
**Summer**	11(15.1)	6(20.7)	7(17.1)				
**Autumn**	22(30.1)	8(27.6)	12(29.3)				
**Winter**	26(35.6)	10(34.5)	13(31.7)	0.993	-	-	-
**Reasons for admission** **[n, (%)]**							
**Cerebrovascular accident**	20(27.4)	7(24.1)	10(24.4)				
**Lung disease** ^**a**^	16(21.9)	11(37.9)	9(22.0)				
**Heart failure** ^**b**^	5(6.8)	2(6.9)	4(9.8)				
**Metabolic disorders**^**c**^	3(4.1)	2(6.9)	7(17.1)				
**Digestive system disease** ^**d**^	4(5.5)	1(3.4)	2(4.9)				
**Cancer**	13(17.8)	2(6.9)	2(4.9)				
**Trauma**	7(9.6)	3(10.3)	6(14.6)				
**Others**	5(6.8)	1(3.4)	1(2.1)	0.406	-	-	-
**Admission APACHE II score(mean ± SD)**	17.58 ±5.588	17.17 ±5.819	16.83 ±3.840	0.754	-	-	-
**Previous antibiotic use**	32(43.8)	10(34.5)	10(24.7)	0.114	-	-	-
**Length of ICU stay before bacteria detected (median, range)**	14(3–31)	14(5–30)	15(4–35)	0.671	-	-	-
**Use of mechanical ventilator [n, (%)]**	54(74.0)	21(72.4)	22(53.7)	0.070	-	-	-
**Infection or colonization**							
**Infection**	32(43.8)	14(48.3)	10(24.4)				
**Colonization**	41(56.2)	15(51.7)	31(75.6)	0.066	-	-	-
**Mortality**	11(15.0)	2(6.9)	3(7.3)	0.323	-	-	-

SD, standard deviation; APACHE, acute physiology and chronic health evaluation; ICU, intensive care unit

^a^Lung disease included patients with acute respiratory failure or pneumonia

^b^ Heart failure included patients with heart attack or acute exacerbation of CHF

^c^ Metabolic disorders included patients with diabetes mellitus ketoacidosis.

^d^ Digestive system disease included patients with hemorrhage of digestive tract, ileac pssion, variceal bleeding, hepatitis, cholecystitis or pancreatitis.

The antimicrobial susceptibility test results are listed in Table 2. *A*. *baumannii* was significantly less susceptible than *A*. *nosocomialis* and *A*. *pittii* to gentamicin, amikacin imipenem, meropenem, doripenem, ciprofloxacin, levofloxacin, piperacillin/tazobactam, ticarcillin/clavulanate, ceftazidime, cefepime, trimethoprim/sulphamethoxazole, ampicillin/sulbactam, and tigecycline (*p* < *0*.001) but not colistin (*p* = 0.673). Rates of multidrug resistance among *A*. *baumannii* isolates were significantly (*p* < 0.001) higher than among *A*. *nosocomialis* and *A*. *pittii* isolates.

**Table 2:** Comparison of antimicrobial susceptibility among different genospecies of ACB complex.

	Susceptibility, n (%)				Statistical analysis (*P* value)			
	A. baumannii n = 73, n (%)	A. nosocomialis, n = 29 n (%)	A. pittii, n = 41 n (%)		Overall	A. baumannii versus A. nosocomialis	A. nosocomialis versus A. pittii	A. baumannii versus A. pittii
**Antibiotics**								
**Gentamicin**	16(21.9)	23(79.3)	30(73.2)		<0.001	<0.001	0.555	<0.001
**Amikacin**	16(21.9)	24(82.8)	35(85.4)		<0.001	<0.001	1.000	<0.001
**Imipenem**	18(24.7)	25 (86.2)	38(92.7)		<0.001	<0.001	0.627	<0.001
**Meropenem**	18(24.7)	25(86.2)	38(92.7)		<0.001	<0.001	0.627	<0.001
**Doripenem**	17(23.3)	25(86.2)	38(92.7)		<0.001	<0.001	0.627	<0.001
**Ciprofloxacin**	15(20.5)	23(79.3)	35(85.4)		<0.001	<0.001	0.734	<0.001
**Levofloxacin**	16(21.9)	24(82.8)	35(85.4)		<0.001	<0.001	1.000	<0.001
**Piperacillin/tazobactam**	16(21.9)	21(72.4)	30(73.2)		<0.001	<0.001	0.944	<0.001
**Ticarcillin/clavulanate**	22(30.1)	24(82.8)	30(73.2)		<0.001	<0.001	0.347	<0.001
**Ceftazidime**	16(21.9)	21(72.4)	29(70.7)		<0.001	<0.001	0.878	<0.001
**Cefepime**	15(20.5)	22(75.9)	30(73.2)		<0.001	<0.001	0.800	<0.001
**Trimethoprim/sulphamethoxazole**	23(31.5)	24(82.8)	30(73.2)		<0.001	<0.001	0.347	<0.001
**Ampicillin/sulbactam**	15(20.5)	20(69.0)	38(92.7)		<0.001	<0.001	0.023	<0.001
**Colistin**	71(97.3)	29(100)	40(97.6)		0.673	-	-	-
**Tigecycline**	43(58.9)	27(93.1)	40(97.6)		<0.001	<0.001	0.758	<0.001
**Resistance profiles**								
**Multidrug resistance**	63(86.3)	6(20.7)	9(22.0)		<0.001	<0.001	0.899	<0.001

Table 3 shows the results of the univariate and multiple variable logistic regression analyses of infection risk factors for infection versus colonization patients with ACB complex isolated from tracheal secretions. Based on the univariate analysis, age, length of stay in the ICU, APACHE II score, use of a mechanical ventilator, and previous antibiotic use were introduced for multiple variable logistic regression analysis (*p* < 0.1). The independent risk factors for infection identified by the multiple variable logistic regression analysis included age [odds ratio (OR) = 1.032, 95% confidence interval (CI) = 1.002–1.063; *p* = 0.039], length of ICU stay (OR = 1.167, 95% CI = 1.071–1.272; *p* < 0.001), APACHE II score (OR = 1.130, 95% CI = 1.010–1.265; *p* < 0.001), use of a mechanical ventilator (OR = 63.124, 95% CI = 12.721–313.227; *p* < 0.001), and previous antibiotic use (OR = 33.372, 95% CI = 8.253–134.947; *p* < 0.001). Table 4 shows the results of the univariate and multiple variable logistic regression analyses of risk factors associated with 30-day mortality in patients with ACB complex isolated from tracheal secretions. Based on the univariate analysis, age, infection, APACHE II score, use of a mechanical ventilator, previous antibiotic use, and multidrug resistance were introduced for multiple variable logistic regression analysis (*p* < 0.1). The independent risk factors for 30-day mortality identified by the multiple variable logistic regression analysis included only APACHE II score (OR = 3.387, 95% CI = 1.209–9.488; *p* < 0.05).

**Table 3:** Univariate and multiple variable logistic regression analyses of infection risk factors in patients with ACB complex isolated from tracheal secretions.

	Infection, n = 56, n (%)	Colonization, n = 87, n (%)	*P* value	Multivariate analysis		
				OR	95%CI	*P* value
**Age, years(mean ± SD)**	66.79 ±17.626	57.31 ±17.503	0.002	1.032	1.002–1.063	0.039
**Gender**						
**Male**	32(57.1)	56(64.4)				
**Female**	24(42.9)	31(35.6)	0.386	-	-	-
**Seasonal distribution**						
**Spring**	9(16.1)	19(21.8)				
**Summer**	7(12.5)	17(19.5)				
**Autumn**	18(32.1)	24(27.6)				
**Winter**	22(39.3)	27(31.0)	0.475	-	-	-
**Reasons for admission** **[n, (%)]**						
**Cerebrovascular accident**	18(32.1)	19(21.8)				
**Lung disease** ^**a**^	12(21.4)	24(27.6)				
**Heart failure** ^**b**^	3(5.4)	8(9.2)				
**Metabolic disorders**^**c**^	5(8.9)	7(8.0)				
**Digestive system disease** ^**d**^	2(3.6)	5(5.7)				
**Cancer**	8(14.3)	9(10.3)				
**Trauma**	5(8.9)	11(12.6)				
**Others**	3(5.4)	4(4.6)	0.799	-	-	-
**Length of ICU stay, day(median, range)**	17(3–35)	13(4–24)	0.002	1.167	1.071–1.272	<0.001
**APACHE II score(mean ± SD)**	18.79 ±5.966	16.43 ±4.492	0.013	1.130	1.010–1.265	0.033
**Use of mechanical ventilator**	51(91.1)	46(52.9)	<0.001	63.124	12.721–313.227	<0.001
**Previous antibiotic use**	30(53.6)	22(25.3)	0.001	33.372	8.253–134.947	<0.001
**A. baumannii**	32(57.1)	41(47.1)	0.242	-	-	-
**A. nosocomialis**	14(25.0)	15(17.2)	0.260	-	-	-
**Multidrug resistance**	35(62.5)	43(49.4)	0.125	-	-	-

OR, odd ratio; CI, confidence interval; SD, standard deviation; ICU, intensive care unit; APACHE, acute physiology and chronic health evaluation

The *p*-value of Hoshmer-Lemeshow goodness-of-fit test was 0.799> 0.05, thus the model was considered as fit for the multiple variable logistic regression.

**Table 4:** Univariate and multiple variable logistic regression analyses of risk factors associated with 30-day mortality in patients with ACB complex isolated from tracheal secretions.

	Died, n = 16, n (%)	Survived, n = 127, n (%)	Univariate analysis *P* value	Multivariate analysis		
				OR	95%CI	*P* value
**Age, years(mean ± SD)**	81.31 ±13.255	58.46 ±17.006	<0.001	-	-	0.918
**Gender**						
**Male**	11(68.8)	77(60.6)				
**Female**	5(31.3)	50(39.4)	0.529	-	-	-
**Seasonal distribution**						
**Spring**	2(12.5)	26(20.5)				
**Summer**	2(12.5)	22(17.3)				
**Autumn**	5(31.3)	37(29.1)				
**Winter**	7(43.8)	42(33.1)	0.764	-	-	-
**Reasons for admission** **[n, (%)]**						
**Cerebrovascular accident**	4(25.0)	33(26.0)				
**Lung disease** ^**a**^	6(37.5)	30(23.6)				
**Heart failure** ^**b**^	0(0)	11(8.7)				
**Metabolic disorders**^**c**^	1(6.3)	11(8.7)				
**Digestive system disease** ^**d**^	0(0)	7(5.5)				
**Cancer**	3(18.8)	14(11.0)				
**Trauma**	1(6.3)	15(11.8)				
**Others**	1(6.3)	6(4.7)	0.705	-	-	-
**Length of ICU stay, day(median, range)**	13(4–35)	15(3–31)	0.972	-	-	-
**Infection**	10(62.5)	46(36.2)	0.042	-	-	0.998
**APACHE II score(mean ± SD)**	27.34 ±1.972	16.02 ±3.904	<0.001	3.387	1.209–9.488	0.02
**Use of mechanical ventilator**	16(100.0)	81(63.8)	0.003	-	-	0.997
**Previous antibiotic use**	15(93.8)	37(29.1)	<0.001	-	-	0.075
**A. baumannii**	11(68.8)	62(48.8)	0.133	-	-	-
**A. nosocomialis**	2(12.5)	27(21.3)	0.412	-	-	-
**A. pittii**	3(18.8)	38(29.9)	0.352	-	-	-
**Multidrug resistance**	13(81.3)	65(51.2)	0.023	-	-	0.124

OR, odd ratio; CI, confidence interval; SD, standard deviation; ICU, intensive care unit; APACHE, acute physiology and chronic health evaluation

The *p*-value of Hoshmer-Lemeshow goodness-of-fit test was 1.000 > 0.05, thus the model was considered as fit for the multiple variable logistic regression.

## Discussion

Although *A*. *baumannii* is generally the most common genospecies of the ACB complex, with the development of identification methods, *A*. *nosocomialis* and *A*. *pittii* have also been reported to be clinically important [2,4,13]. In our study, the prevalence of *A*. *baumannii*, *A*. *nosocomialis*, and *A*. *pittii* was different from previous reports [14,15], which probably result from differences in the specific epidemiological situations of various centres and in the sites from which bacteria were isolated and detected [16]. In our study, all ACB complex bacteria were isolated from patient tracheal secretions. Not all ACB complex genospecies caused infection. More than half of the ACB complex represented bacterial colonization. This finding differs from those of previous studies [2–5], in which ACB complex genospecies were isolated from blood, and all of them caused infection. A previous study showed that *A*. *nosocomialis* exhibited higher virulence than other species [13, 17]. Some studies have reported that infections caused by *A*. *baumannii* are associated with an increase in mortality [4, 5], possibly due to the high multidrug resistance of *A*. *baumannii*, which invalidates the use of antibiotics. Others have indicated that there is no significant correlation between bacterial infection and an increase in mortality [18, 19, 20], showing that although species differ in terms of virulence, the pathogenicity of the ACB complex is generally low. In our study, the distribution of infection and colonization as well as mortality of the three genospecies were the same. In addition, we found that mortality caused by ACB complex isolated from tracheal secretions was much lower than that isolated from blood in previous studies [2, 3, 5]. This result could be due to the sites of bacteria detection. Different bacterial infection sites may lead to a different progression and outcome of the disease.

Antibiotic resistance among ACB complex bacteria is severe, especially in the ICU [2]. Consistent with the results of a previous study, we found that different genospecies of the ACB complex showed different sensitivities to commonly used antibiotics [2–5]. *A*. *baumannii* exhibited higher rates of antibiotic resistance than the other two genospecies, which may be due to different biological features, such as the ability to adhere to eukaryotic cells or differences in the expression of multidrug resistance genes [16, 17, 21–27].

Consistent with a previous study [28], advanced age, a long stay in the ICU, high APACHE II score, the use of a mechanical ventilator, and previous antibiotic use are risk factors of infection for patients with ACB complex. ACB complex bacteria can easily survive in the environment and on artificial devices. The use of a mechanical ventilator may make patients more vulnerable to infection. Patients in the ICU are threatened by underlying diseases, the accumulation of antibiotics and suppressed immune states that may make them more easily infected after initial colonization [29]. Kuo LC’s study indicated that multidrug resistance of ACB complex bacteria was a risk factor for mortality [30], while Fitzpatrick found that multidrug resistance of ACB complex bacteria was not a risk factor for mortality [3], which was the same as the finding of our study. The limited number of ACB complex isolates collected and the unique site of bacteria detected in our study could be responsible for the observed negative results. Consist with previous studies [17, 20], we found that the severity of patients’ underlying disease is associated with mortality.

Our study had several limitations. First, in terms of research content, we did not have data or analysis on antibiotic treatment. Second, selection bias, information bias and possibility of confounding can occur in retrospective cohort studies.

In conclusion, we found that there were no differences in clinical manifestations and that there were significant differences in antimicrobial susceptibility patterns among patients with different genospecies of ACB complex bacteria isolated from tracheal secretion samples. Advanced age, a long stay in the ICU, high APACHE II score, the use of a mechanical ventilator, and previous antibiotic use were risk factors of infection for ACB complex isolated from the tracheal secretions. High APACHE II score was the risk factor of mortality. As important bacteria in nosocomial infection, further investigation on ACB complex is necessary.

## Supporting information

S1 Table (RAR)

S2 Table (RAR)

S3 Table (JPG)

S4 Table (JPG)
