# Supplementary figures and images for: Comparison of clinical manifestations and antibiotic resistances among three genospecies of the Acinetobacter calcoaceticus-Acinetobacter baumannii complex
Source: PLoS One. 2018 Feb 1;13(2):e0191748. doi: 10.1371/journal.pone.0191748 (PMC5794090; doi:10.1371/journal.pone.0191748)

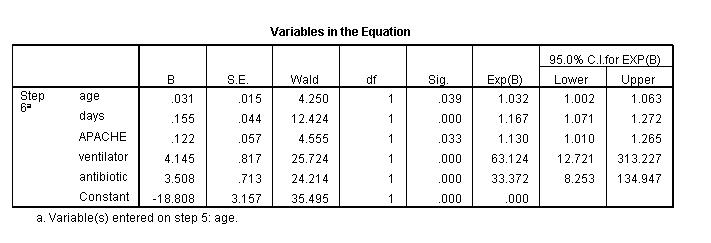

Supplement: S3 Table — (JPG) [file pone.0191748.s003.jpg]

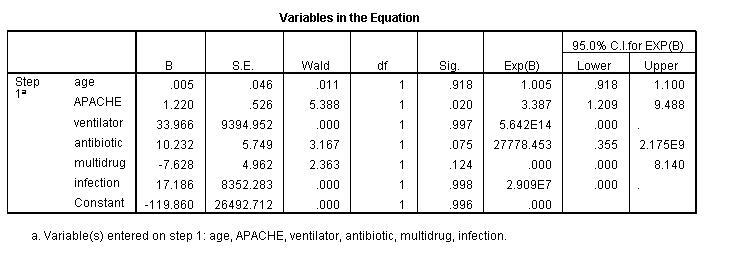

Supplement: S4 Table — (JPG) [file pone.0191748.s004.jpg]
